# Clonidine in the treatment of adolescent chronic fatigue syndrome: a pilot study for the NorCAPITAL trial

**DOI:** 10.1186/1756-0500-5-418

**Published:** 2012-08-07

**Authors:** Even Fagermoen, Dag Sulheim, Anette Winger, Anders M Andersen, Nils Tore Vethe, J Philip Saul, Erik Thaulow, Vegard Bruun Wyller

**Affiliations:** 1 Department of Pediatrics, Oslo University Hospital and University of Oslo, Oslo, Norway; 2 Department of Anesthesiology, Oslo University Hospital and University of Oslo, Oslo, Norway; 3 Lillehammer County Hospital, Lillehammer, Norway; 4 Department of Nursing Education, Oslo University College, Oslo, Norway; 5 Department of Pharmacology, Oslo University Hospital, Oslo, Norway; 6 Department of Medical Biochemistry, Oslo University Hospital, Oslo, Norway; 7 Department of Pediatrics, Medical University of South Carolina, Charleston, SC, USA; 8 Department of Paediatrics, Oslo University Hospital, P.O. Box 4950, Nydalen 0424, Oslo, Norway

**Keywords:** Chronic fatigue syndrome, Adolescents, Clonidine, Adverse effects, Head-up tilt test, Autonomic nervous system

## Abstract

**Background:**

This pilot study (ClinicalTrials.gov ID: NCT01507701) assessed the feasibility and safety of clonidine in adolescent chronic fatigue syndrome (CFS). Specifically, we assessed clonidine dosage in relation to a) plasma concentration levels, b) orthostatic cardiovascular responses, and c) possible adverse effects.

**Findings:**

Five adolescent CFS patients (14–19 years old) received 50 μg clonidine twice per day during 14 days in an open, uncontrolled design. Plasma concentration of clonidine was assayed by standard laboratory methods. Changes in orthostatic cardiovascular responses were assessed by a 20^o^ head-up tilt-test (HUT). Adverse effects were mapped by a questionnaire.

After 14 days, C_0_ median (range) of clonidine was 0.21 (0.18-0.36) μg/L, and C_max_ median (range) of clonidine was 0.41 (0.38-0.56) μg/L. Also, supine blood pressures and heart rate were lower during clonidine treatment, and the HUT response was closer to the normal response. No serious adverse effects were registered.

**Conclusion:**

Clonidine 50 μg BID seems to be safe enough to proceed from a pilot study to a controlled trial in a select group of adolescents with CFS (ClinicalTrials.gov ID: NCT01040429).

## Findings

### Background

Chronic Fatigue Syndrome (CFS) is a relatively common and disabling condition among adolescents in developed countries [1,2]. A study from 2004 reports the prevalence among 8–17 years olds as high as 1.3 % [3]. The level of disability varies, but a majority has long-lasting absence from school, and is unable to participate in normal social activities. The most disabled are permanently bedridden.

The pathophysiology of CFS remains poorly understood. However, previous reports have documented enhanced sympathetic nervous activity at rest and during moderate somatic stressors, affecting cardiovascular control [4]. For instance, previous experiments at our institution have shown higher blood pressures, heart rate and LF/HF ratio (low-frequency:high-frequency heart rate variability ratio, an index of sinus node sympathovagal balance derived from spectral analyses of heart rate) at rest among adolescent CFS patients as compared to healthy controls, and a stronger increase in these variables upon orthostatic stress, such as head-up tilt [5-7]. A detailed study of the baroreceptor reflex suggests alterations of the central “set-point” [8]. Similarly, ambulatory measurements of blood pressures and heart rate indicate higher nocturnal values among CFS patients as compared to controls [9], and studies of neuroendocrinology report higher levels of catecholamines [10]. Taken together, these findings suggest that enhanced sympathetic nervous activity due to functional alteration in the central nervous system might constitute a key element of CFS pathophysiology and might be directly related to clinical symptoms and disability [11,12]. Alternatively, the autonomic alterations in CFS might be secondary to another disease mechanism, such as hypovolemia [13]. However, important characteristics of the cardiovascular responses among CFS patients does not support this possibility, as has been thoroughly discussed elsewhere [8,11].

To date, only cognitive behavioral therapy and graded exercise therapy have a well-documented beneficial effect in the treatment of CFS [2,14]. Thus, searching for a pharmacological therapy should have high priority, and the documented enhancement of sympathetic nervous activity represents a possible target for intervention. Clonidine is an agonist to the adrenergic, inhibitory α_2_-receptor. It has a general inhibitory influence on sympathetic nervous activity, in particular due to its effect in the central nervous system, resulting in lower blood pressures and heart rate [15]. Therefore, clonidine might be a potentially useful therapy in CFS. To our knowledge, no prior study of clonidine in CSF has been undertaken. In a trial of this drug, the well-known alteration of cardiovascular control in CFS patients might serve as a biomarker.

An area of concern, however, is the possibility of adverse effects. In fact, clonidine might worsen rather than improve the orthostatic intolerance among CFS patients, increasing the risk of syncope; this is particularly true if the autonomic alterations are secondary to hypovolemia rather than a primary disease mechanism. Also, CFS patients are generally considered to be sensitive towards adverse pharmacological effects, maybe because of altered pharmacodynamics [14]. Therefore, a pilot study assessing feasibility and safety of clonidine therapy is necessary prior to a large-scale trial. Of note, recent randomized controlled trials reported a beneficial effect of clonidine and low risk of side-effects in pediatric attention deficit/hyperactivity disorder (AD/HD) and tic disorders [16,17]. Also, in pediatric practice, clonidine is used for analgesic purposes, to alleviate abstinence responses, and against spasticity [18,19].

The Norwegian Study of Chronic Fatigue Syndrome in Adolescents: Pathophysiology and Intervention Trial (NorCAPITAL) is a randomized, placebo-controlled, double-blind trial of clonidine in adolescent CFS (ClinicalTrials.gov ID: NCT01040429). The aim of this pilot study (ClinicalTrials.gov ID: NCT01507701) was to investigate the feasibility and safety of clonidine in adolescent CFS. Specifically, we wanted to assess appropriate dosage in relation to a) plasma concentration levels, b) orthostatic cardiovascular responses, and c) reports of possible adverse effects, in particular syncope.

### Patients

Five adolescent CSF patients were recruited from the Pediatric outpatient clinic, Oslo University Hospital, Rikshospitalet, Norway, which serves as a national referral center for children and adolescents with unexplained chronic fatigue. Other disease states that might explain their present symptoms, such as autoimmune, endocrine, neurologic or psychiatric disorders (including depression and anxiety), were ruled out by a thorough and standardized set of investigations.

Different case definitions of CFS exist. The frequently used definition from the International Chronic Fatigue Syndrome Study Group (commonly referred to as the Fukuda-definition) requires at least six months of unexplained chronic or relapsing fatigue of new onset, severely affecting daily activities, as well as four or more of eight specific accompanying symptoms (headache, muscle pain, joint pain, sore throat, tender lymph nodes, impaired memory or concentration, unrefreshing sleep, and malaise after exertion) [20]. The validity of this definition has been questioned [21]. Therefore, in this study, three consecutive months of unexplained disabling fatigue and no accompanying symptoms were required for inclusion. This approach is in line with the clinical recommendations from The Royal College of Paediatrics and Child Health [2] and the National Institute for Health and Clinical Excellence [14], and is also proven feasible in previous studies from our group [5,10].

Exclusion criteria included supine systolic blood pressure (SBP) < 85 mm Hg, a fall in SBP upon standing > 30 mm Hg, supine heart rate (HR) < 50 beats/min, abnormal ECG, permanent bed-rest, regular use of pharmaceuticals, other chronic diseases and a positive pregnancy test.

### Study protocol

Patients were invited to two clinical encounters at our institutions, separated by a time period of 14 days. At the first encounter, a head-up tilt-test was performed. The subjects were positioned horizontally on a-table with foot-board support (Model 900–00, CNS-systems Medizintechnic, Graz, Austria) and attached to the Task Force Monitor® (Model 3040i, CNSystems Medizintechnic, Graz, Austria), a device for non-invasive recording of cardiovascular variables [22]. We obtained a 5 minute baseline recording before tilting head-up to 20° over 10 seconds and maintaining that position for 15 minutes, followed by another 5 minutes epoch in the horizontal position. Instantaneous heart rate (HR) was obtained from the R-R interval of the electrocardiogram; each R deflection was automatically detected by the Task Force Monitor software. In addition, the RR-interval was subjected to spectral analysis using an adaptive autoregressive algorithm, calculating spectral power densities in the low-frequency (LF) band (0.04-0.15 Hz) and the high-frequency (HF) band (0.15-0.4 Hz). These indices are measures of autonomic heart rate control; the LF/HF-ratio is a measure of “sympathovagal balance”, where higher values indicate enhanced sympathetic activity. Photoplethysmography on the right middle finger was used to obtain a non-invasive, continuous recording of arterial blood pressure, and impedance cardiography was used to obtain a continuous recording of the stroke volume. All recorded signals were on-line transferred to the built-in recording computer of the Task Force Monitor®, running software for real-time data acquisition. Immediately after the tilt test, subjects were given 50 μg clonidine orally. During the next 5 hours, they were monitored with repeated blood samples for clonidine concentration assays.

The participants were instructed to take 50 μg clonidine twice per day (BID) until the second clinical encounter, which consisted of blood samples (for assays of clonidine concentration) and a repeated head-up tilt test. Also, participants were questioned about possible side-effects of clonidine treatment (such as sleepiness, constipation, dizziness, syncope and dry mucus membranes). At discharge from the second clinical encounter, they were instructed to take 25 μg clonidine BID for another week, after which the treatment was discontinued.

Two days in advance of both encounters, all participants were instructed not to drink beverages containing alcohol or caffeine, not to take any drugs, and not to use tobacco products. They were requested to fast overnight prior to the first clinical encounter, and apply an ointment of the local anesthetic lidocaine (Emla®) in the antecubital fossa one hour before venous puncture. Blood samples and HUT were carried out between 8 and 10 am at both occasions in a quiet, warm room.

Written, informed consent was obtained from all participants and their parents. The study was approved by the Regional committee for ethics in medical research (REK Sør-Øst C) and The Norwegian Medicines Agency.

### Laboratory procedures

The blood samples for clonidine determinations were collected in 4 mL heparin tubes. After centrifugation for 12 minutes at 1000 g at room temperature, the plasma fraction was frozen at −20°C until analysis within 2 weeks. Plasma concentrations of clonidine were assayed by the method of Müller *et al.* with some modifications [23]. The samples were separated on an Alliance HT 2795 HPLC system and detected by a Micromass Quattro micro API MS/MS-instrument (Waters, Milford, MA). The MS/MS conditions were optimized by manual tuning during pump-infusion of neat solutions. Due to the low dose of clonidine used in this study, the high end of the calibration range was omitted. Calibrated range was from 0.10 μg/L to 5.00 μg/L. The intra assay CV was median 1.0 % at 5.00 μg/L and median 10 % at 0.10 μg/L (lower limit of quantification).

### Data analyses

All data were exported to Microsoft Excel for further analyses. From each experimental run of HUT, we calculated the median of all variables in two epochs: From 270 to 30 seconds prior to tilt (Baseline) and from 30 to 270 seconds after beeing tilted (Tilt). We also computed delta values (Tilt – Baseline). Cardiac output was computed as stroke volume times HR, whereas total peripheral resistance was calculated as mean arterial blood pressure (MBP) divided by cardiac output. Relevant hemodynamic variables were indexed against body surface area. This method of analyzing results from HUT has successfully been used in previous projects from our institution [5,6].

Results are displayed as median and total range. Due to the low number of participants, we did not carry out statistical tests.

### Results

Five patients (three males and two females, median age 14 years, median disease duration 34 months) were screened; they all adhered to the inclusion and exclusion criteria, and were therefore enrolled in the project (Table 1). All patients had a high level of school absenteeism, but no one was permanently bed-ridden.

**Table 1:** Population characteristics and clonidine concentration at steady state

Number (male/female)	5 (3/2)
	*Median (range)*
Age (years)	14
	(14–19)
BMI (kg/m^2^)	20.8
	(17.0-22.4)
Disease duration (months)	34
	(27–48)
C_0_ of clonidine (μg/L),	0.21
	(0.18-0.36)
C_max_ of clonidine (μg/L)	0.41
	(0.38-0.56)

After the initial, single oral dose of 50 μg clonidine, plasma concentration levels rose to a maximum level after 60 – 120 minutes (T_max_) in all patients; median (range) value was 0.26 (0.14 - 0.29) μg/L. After 14 days of clonidine treatment, median trough concentration (C_0_) was 0.21 μg/L, rising to a median level of 0.41 μg/L (C_max_) two hours after administration of the regular dose (Table 1).

The head up tilt test showed a slight reduction in median HR, MBP, DBP and SI at baseline during clonidine treatment (Table 2). At tilt, blood pressures tended to decrease rather than increase during clonidine treatment, whereas the increase in TPRI and LF/HF was less pronounced.

**Table 2:** Hemodynamic variables before and during clonidine treatment. Median (range) values at supine rest (baseline) and the response (Δ) to 20 deg. head-up tilt-test

	***Baseline***		***Δ (Tilt - Baseline)***	
	**Before clonidine**	**During clonidine**	**Before clonidine**	**During clonidine**
HR (beats/min)	69.5	65.4	1.7	1.4
	(63.1-107.1)	(59.5-88.0)	(−0.1-4.4)	(−1.0-6.7)
SBP (mm Hg)	101.8	101.2	0.9	−1.5
	(91.0-104.9)	(96.2-107.4)	(−10.4-5.1)	(−5.7-9.0)
MBP (mm Hg)	76.1	74.0	2.5	−2.0
	(65.4-76.1)	(68.9-80.8)	(−9.5-5.6)	(−3.7-2.0)
DBP (mm Hg)	62.1	59.1	4.0	−2.8
	(53.5-70.6)	(57.4-64.0)	(−9.4-4.6)	(−3.2-0.8)
SI (ml/m^2^)	50.4	42.7	−4.8	−3.4
	(32.1-62.2)	(34.6-78.2)	(−6.0--2.8)	(−8.2--1.3)
CI (l/min/m^2^)	3.4	3.0	−0.3	−0.1
	(3.3-3.9)	(2.5-4.7)	(−0.3-0.0)	(−0.4-0.0)
TPRI (mm Hg/l/min/m^2^)	9.0	9.0	0.8	0.3
	(6.3-9.6)	(5.2-13.2)	(−0.3-1.6)	(−0.1-1.1)
LF/HF	0.56	0.57	0.58	0.36
	(0.30-0.93)	(0.38-0.64)	(−0.05-0.82)	(−0.01-0.68)

HR=heart rate; SBP=systolic blood pressure; MBP=mean arterial blood pressure; DBP=diastolic blood pressure; SI=stroke index; CI=cardiac index; TPRI=total peripheral resistance index; LF/HF=low-frequency:high-frequency heart rate variability ratio.

No serious adverse effects were registered during the treatment period (Table 3). Initially, all patients reported a feeling of dry eyes and two reported a feeling of dry mouth; these symptoms subsided after a few days. Three of the patients reported a change in sleeping pattern; one reported lighter sleep, one uneasy sleep, and one better sleep. Also, one patient reported sleepiness at daytime. One patient experienced increased fatigue, and one patient described slight dizziness.

**Table 3:** Reported adverse effects of clonidine during the treatment period

	***Dry mucus membranes (eyes/mouth)***	***Lighter/uneasy sleep***	***Heavier sleep***	***Sleepiness at daytime***	***Increased fatigue***	***Dizziness***
Patient #1	x	x			x	
Patient #2	x			x		
Patient #3	x	x				
Patient #4	x					x
Patient #5	x		x			

### Discussion

This pilot study indicates that 50 μg clonidine BID might be safe in the treatment of adolescent CFS. The plasma concentration levels were adequate, the orthostatic responses were closer to the normal responses, and the therapy was well tolerated by the patients with no serious side effects.

The pharmacokinetics and antihypertensive effects of low dose clonidine treatment have been studied among adult hypertensive patients [24]. Applying a dosage of 75 μg clonidine bd during 5 days, mean C_max_ and T_max_ were reported to be 0.65 μg/L and 2.5 hrs, respectively, corresponding neatly with our results. Also, a slight, but significant antihypertensive effect was found, in line with our data. Other studies report that a plasma concentration of 0.2 – 2.0 μg/L of clonidine is associated with clinical effects [25,26]; in this study, the plasma concentrations of clonidine in all individuals were within this range, but in the lower end.

In CFS patients, orthostatic challenges cause a stronger increase in HR, blood pressures, TPRI and LF/HF as compared to healthy controls [5,6]. In this study, after clonidine treatment, blood pressure values tended to decrease rather than increase, and the increase in TPRI and LF/HF was less pronounced. Thus, clonidine seems to normalize the head-up tilt-test responses. Similar results were found in adult patients with ulcerative colitis treated with clonidine [15]. Previously, we have suggested that the abnormal cardiovascular responses during orthostatic challenges in CFS patients are connected with a key feature of the underlying pathophysiology [11]. We therefore speculate that clonidine not only might improve cardiovascular control, but also might have a beneficial effect on fatigue and functional ability in adolescent CFS. This hypothesis should be tested in a standard randomized controlled trial, in which the head-up tilt-test might serve as a biomarker.

The patients’ reports of dry mucus membranes, altered sleeping pattern, and slight dizziness are well known side-effects of clonidine [27]. However, sleeping difficulties and dizziness might also represent natural fluctuation of CFS symptom intensity, and should not automatically be ascribed to clonidine. Similarly, increased fatigue – reported by one patient – might represent an adverse effect which is of particular concern in patients with CFS; however, increased fatigue might equally well stem from alterations of disease activity. These uncertainties should be specifically addressed in a randomized controlled trial. Given the orthostatic intolerance of CFS, we were concerned that clonidine treatment might provoke syncope in this patient group. However, syncope was not experiences by any of the participants in this study, nor was there any large reduction of blood pressures during tilt. The low dosages, yielding plasma concentration within the lower range of what is considered clinically effective, probably contributed to low incidence of side-effects. This might be particularly important in CFS patients, which are often considered to be particularly sensitive towards adverse pharmacological effects [14].

### Study limitations

We did not estimate the frequency of syncope or other adverse effects prior to this study; nor did we perform a power calculation. To the best of our knowledge, clonidine has never been subjected to a clinical trial in CFS patients; therefore, instead of assuming anything about effects, we found it more appropriate to conduct a small-scale pilot study. Hypovolemia remains an alternative explanation for the autonomic alterations among CFS patients [13]; thus measuring blood volume prior to initiation of clonidine would have would have been informative and also improved study safety. Generally, the small number of participants and the open-label, uncontrolled design of this study do not allow firm conclusion.

### Conclusions

Clonidine 50 μg BID seems to be safe enough to proceed from a pilot study to a controlled trial in a select group of adolescents with CFS (ClinicalTrials.gov ID: NCT01040429).

## Abbreviations

AD/HD, Attention deficit/Hyperactivity disorder; CFS, Chronic fatigue syndrome; CI, Cardiac index; DBP, Diastolic blood pressure; HF, High-frequency heart rate variability; HR, Heart rate; HUT, Head-up tilt-test; LF, Low-frequency Heart Rate Variability; LF/HF, Low-frequency: high-frequency heart rate variability ratio; MBP, Mean arterial blood pressure; NorCAPITAL, The Norwegian study of chronic fatigue syndrome in adolescents: pathophysiology and intervention trial; SBP, Systolic blood pressure; SI, Stroke index; TPRI, Total peripheral resistance index.

## Competing interests

The authors declare that they have no competing interests.

## Authors’ contributions

EF, DS and AW carried out the clinical investigations, head-up tilt-tests and data analyses. EF drafted the manuscript; DS and AW helped to draft the manuscript. AMA and NTV carried out the laboratory investigations and helped to draft the manuscript. JPS, ET participated in study planning and design, and helped to draft the manuscript. VBW conceived of the study, participated in its design and coordination and helped to draft the manuscript. All authors have read and approved the final version of the manuscript.
